# Novel High-Capacitance-Ratio MEMS Switch: Design, Analysis and Performance Verification

**DOI:** 10.3390/mi9080390

**Published:** 2018-08-06

**Authors:** Ke Han, Xubing Guo, Stewart Smith, Zhongliang Deng, Wuyu Li

**Affiliations:** 1 School of Electronic Engineering, Beijing University of Posts and Telecommunications, Haidian District, Beijing 100876, China; gxb@bupt.edu.cn (X.G.); dengzhl@bupt.edu.cn (Z.D.); liwuyu@bupt.edu.cn (W.L.); 2 School of Engineering, University of Edinburgh, Edinburgh EH9 3FF, UK; stewart.smith@ed.ac.uk

**Keywords:** RF MEMS, high-capacitance-ratio, metal-insulator-metal

## Abstract

This paper proposes a novel high-capacitance-ratio radio frequency micro-electromechanical systems (RF MEMS) switch. The proposed RF MEMS mainly consists of serpentine flexure MEMS metallic beam, comprised of coplanar waveguide (CPW) transmission line, dielectric and metal-insulator-metal (MIM) floating metallic membrane. Comparing the proposed high-capacitance-ratio MEMS switch with the ones in available literature, an acceptable insertion loss insulation, acceptable response time and high capacitance ratio (383.8) are achieved.

## 1. Introduction

In today’s competitive wireless market, compact, low cost, reconfigurable, multiband solutions are required and reconfigurable radio frequency (RF) blocks is the research hotspot. The radio frequency micro-electromechanical systems (RF MEMS) switch draws considerable attention owing to its attractive performance, as a key enabler for reconfigurable RF front-ends [1,2]. Compared with conventional RF switches (variodes, PIN diodes, and other technologies), RF MEMS switches possess many attractive advantages, such as high linearity, high quality factors, and almost no DC power consumption.

However, there are three main problems usually confronted by research: low capacitance ratio (capacitive shunted switch), high actuating voltage and dielectric charging issue [3]. A larger off/on capacitance ratio is beneficial to high isolation performance, low actuating voltage contributes to the monolithic microwave integrated circuit (MMIC) implementation and dielectric charging causes failure of RF MEMS switch. In the application of the tunable filter, the capacitance ratio of the switch determines the adjustable range of the center frequency of the resonant unit in the tunable filter. The high off/on capacitance ratios of MEMS switches must be a focus in order to obtain better RF performance.

To date, there are some studies that have been done in larger capacitance off/on ratio and low actuating voltage. Reference [4] proposed a larger capacitance ratio MEMS switch using high dielectric constant ceramics, the high capacitance ratio also was achieved in Reference [5] by employing warped-beam. The other method of high off/on capacitance ratio implementation is enlarging the gap between MEMS beam and dielectric layer [6,7]. However, the capacitance ratio is limited by the minimum the thickness of dielectric layer, the maximum value of the dielectric constant and the maximum gap between beam and signal transmission line. In addition, the dielectric charging issue is serious when the thin dielectric layer is used. Therefore, the methods employed in the [5,6,7] are not the most appropriate.

This paper proposes a novel high-capacitance-ratio MEMS switch based on the floating metal membrane concept, without the restrictions of minimum thickness of dielectric layer and the minimum gap between beam and signal transmission line. The low actuating voltage was also achieved owing to the serpentine flexure MEMS beam.

## 2. Design of the High OFF/ON Capacitance Ratio RF MEMS Switch

**Figure 1:**
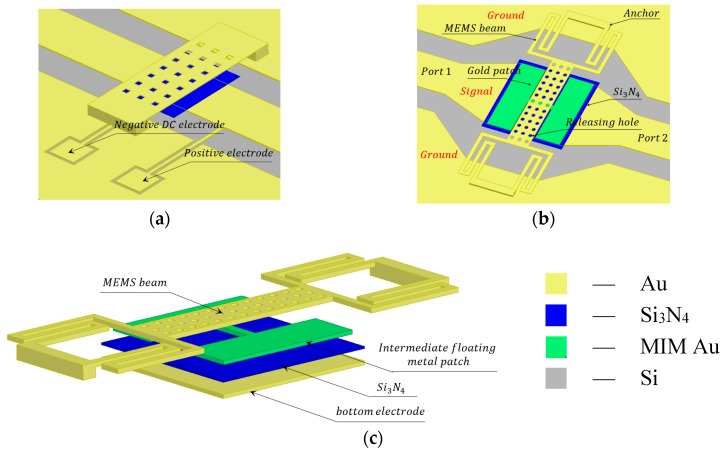
Designed high-capacitance-ratio MEMS switch model. (**a**) Conventional MEMS switch; (**b**) The top view of the proposed MEMS switch; (**c**) The dismantling figure of the MEMS switch. (The gold material is illustrated by yellow and green for the sake of representing different layers.)

The conventional MEMS switch is comprised of coplanar waveguide (CPW) transmission line, metallic beam, dielectric and DC actuating electrodes as shown in Figure 1a. The metallic beam is suspended over CPW with air gap *g_0_*. When the DC voltage is applied to the DC electrodes, electrostatic force pulls the metallic beam down, the RF signal will be cut by this MEMS metallic beam.

A simple and effective approach to obtain a high capacitance ratio of a MEMS switch is to connect a capacitor to the shunt capacitor. The capacitor located on one side of the ground plane of CPW, was proposed in Reference [8].

The proposed novel high-capacitance-ratio MEMS switch is shown in Figure 1b,c. It consists of serpentine flexure MEMS metallic beam, CPW transmission line, dielectric and metal-insulator-metal (MIM) floating metallic membrane. (DC actuating electrodes are not shown in the figures).

**Figure 2:**
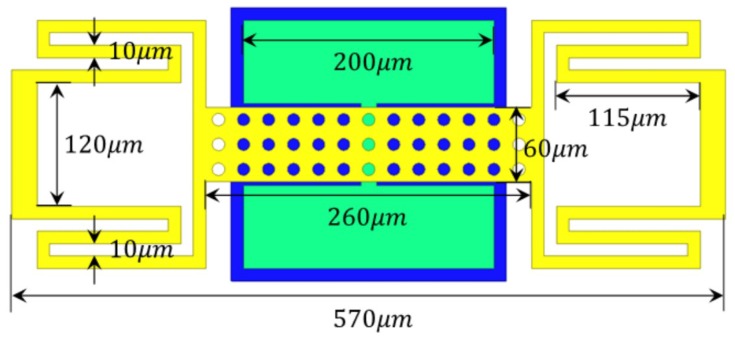
The geometry configuration of the proposed RF MEMS switch.

The geometry configuration of the proposed RF MEMS switch is shown in Figure 2. The RF MEMS switch beam attached with four serpentine springs.

**Figure 3:**
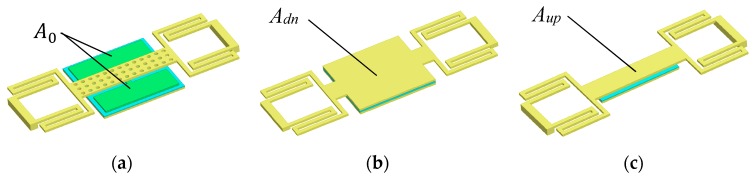
Models of the proposed switch. (**a**) 3D model; (**b**) Equivalent model of down state; (**c**) Equivalent model of up state (some details were neglected for simplification).

A 3D model of the proposed MEMS switch is shown in Figure 1c and Figure 3a. When the MEMS switch is in the down state, the serpentine flexure MEMS metallic beam will contact with the floating metal membrane, the area of the overlapping electrode area will be enlarged as shown in Figure 3b. On the contrary, the serpentine flexure MEMS metallic beam will detach from the floating metallic membrane. As a consequence, the overlapping electrode area will decrease. The constant capacitance $C_{0}$ are formed between MIM floating metallic membrane and signal line. Therefore, the constant capacitance $C_{0}$ and difference of overlapping electrode area will lead to high $C_{off}/C_{on}$ ratio.

## 3. Analysis of High OFF/ON Capacitance Ratio RF MEMS Switch

### 3.1 Restriction Factors of the Conventional RF MEMS Off-to-On Capacitance Ratio

As shown in Figure 1a, for the conventional RF MEMS, the up state (namely ON state) capacitance $C_{on}$ and the down state (namely OFF state) capacitance $C_{off}$ are respectively expressed as follows:

(1) $$\left\{ \begin{array}{l} C_{on}=\varepsilon_{0}A_{up}{\left(g_{0}+\frac{t_{e}}{\varepsilon_{r}}\right)}^{-1}\\ C_{off}=\varepsilon_{0}A_{dn}{\left(\frac{t_{e}}{\varepsilon_{r}}\right)}^{-1} \end{array}\right.$$

where $\varepsilon_{0}$ is dielectric constant in the free space, *g*_0_ is initial air gap between RF MEMS switch beam and the Si_3_N_4_ dielectric when no actuating voltage is applied to the beam, $\varepsilon_{r}$ is relative dielectric constant of Si_3_N_4_ dielectric, *t_e_* is the thickness of Si_3_N_4_ dielectric layer, *A_up_* and *A_dn_* are the overlapping electrode area of up and down state, respectively. Hence, when the fringe effect is neglected, the off-to-on capacitance ratio of conventional RF MEMS switch can be expressed as:

(2) $$r_{c}=\frac{C_{off}}{C_{on}}=\frac{A_{dn}}{A_{up}}\left(1+g_{0}\frac{\varepsilon_{r}}{t_{d}}\right)$$

For the conventional RF MEMS switch, the *A_up_* and *A_dn_* were constant after the switch was fabricated. Therefore, the capacitance ratio is limited by three factors, namely: (a) the relative dielectric constant $\varepsilon_{r}$; (b) the thickness of dielectric *t_e_*; (c) the initial gap *g_0_*. These limiting factors are not resolved easily. First, when the fabrication process is determined, the relative dielectric constant $\varepsilon_{r}$ is determined as well; second, the dielectric charging issue is serious when the thin dielectric layer is used; third, the larger initial gap *g*_0_ will cause high actuating voltage. Hence, the methods used in [5,6,7] are not the most appropriate as mentioned in Section 1.

### 3.2 The High OFF/ON Capacitance Ratio of the Proposed RF MEMS Switch

**Figure 4:**
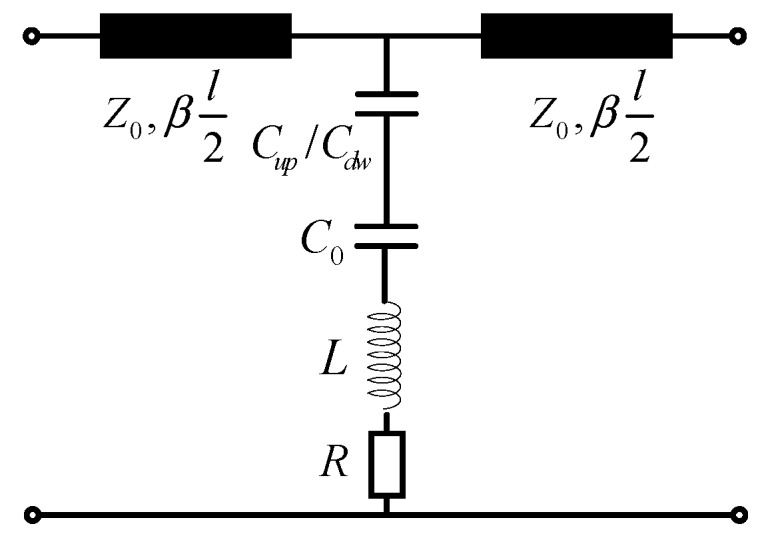
The geometry configuration of the proposed RF MEMS switch.

The model and structure of the proposed RF MEMS switch is shown in Figure 1, and the equivalent circuit model is shown in Figure 4 [9]. The wave port of the characteristic impedance $Z_{0}$ denotes the characteristic impedance of the transmission line between the wave port and the edge of the MEMS beam. *β*$\frac{l}{2}$ denotes electric length of the transmission line. The constant capacitance $C_{0}$ is introduced when the RF MEMS switch is in the up state (namely ON state). However, the $C_{0}$ does not exist when it is in the down state (namely OFF state), for the MIM floating metallic membrane is a part of the *A_dn_* when it is in the down state. Hence, the capacitance $C_{0}$, $C_{on}$ and $C_{off}$ are respectively expressed as:

(3) $$\left\{ \begin{array}{l} C_{on}=\frac{C_{0}\varepsilon_{0}\varepsilon_{r}A_{up}}{\varepsilon_{0}\varepsilon_{r}A_{up}+C_{0}\left(g_{0}\varepsilon_{r}+t_{e}\right)}\\ C_{off}=\frac{\varepsilon_{0}\varepsilon_{r}A_{dn}}{t_{e}}\\ C_{0}=\varepsilon_{0}A_{0}{\left(\frac{t_{e}}{\varepsilon_{r}}\right)}^{-1} \end{array}\right.$$

The capacitance ratio $r_{c}$ of the proposed RF MEMS switch is:

(4) $$r_{c}=\frac{A_{dn}\left[\varepsilon_{0}\varepsilon_{r}A_{up}+C_{0}\left(g_{0}\varepsilon_{r}+t_{e}\right)\right]}{t_{e}C_{0}A_{up}}$$

**Figure 5:**
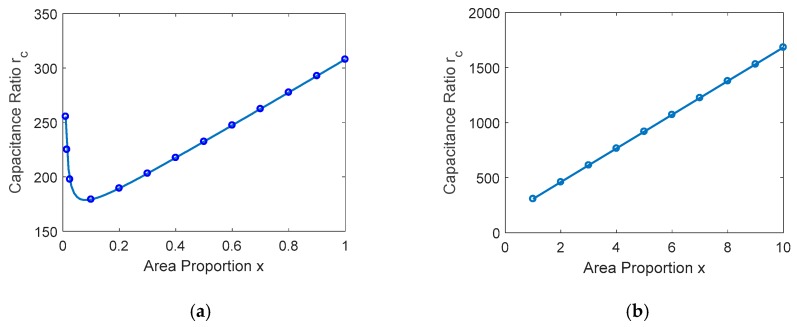
The relationship between x and $r_{c}$. (**a**) x∈[0,1]; (**b**) x∈[1,10].

Assuming the electrode area of MIM floating metallic membrane $A_{0}$ is x times to the $A_{up}$. Hence, the $A_{dn}$ is (x+1) times to the $A_{up}$, namely $A_{0}=xA_{up}$, $A_{dn}=\left(x+1\right)A_{up}$. The capacitance ratio $r_{c}$ is deduced as:

(5) $$r_{c}\left(x\right)=\frac{x+1}{x}\left[1+x\left(\frac{g_{0}\varepsilon_{r}}{t_{e}}+1\right)\right]$$

where $\varepsilon_{0}$ is dielectric constant in the free space, namely 8.85 × 10^−12^ F/m, $\varepsilon_{r}$ is relative dielectric constant, which depends on the dielectric material. According to the equation, the capacitance ratio $r_{c}$ is related to the electrode area ratio x, instead of the specific value of the $A_{0}$, $A_{up}$ and $A_{dn}$. The relationship between x and $r_{c}$ is shown in Figure 5.

Let the $dr_{c}/dx=0$ and arrive at the extreme point $x_{0}$. As shown in Figure 5a, when $x\in \left[0,x_{0}\right]$, the capacitance ratio $r_{c}$ is monotone decreasing. The $r_{c}$ is monotone increasing in the interval $x\in \left[x_{0},+\infty \right]$. When the $\left[x_{0},+\infty \right]$, the resonant frequency of MEMS is decreased due to the increase of the shunt capacitance. Figure 5 provides a theory guide for the design of MEMS switches. In this proposed RF MEMS switch, $\varepsilon_{r}=7.6$, x=2, $t_{e}=1000\;Å$, $g_{0}=2\;\mu m$, $A_{up}=200\times 60{\;\mu m}^{2}$, $A_{dn}=200\times 60\times 3{\;\mu m}^{2}$ and $A_{0}=200\times 60\times 2{\;\mu m}^{2}$. Thus, we arrive at $C_{0}=16.1\;\mathrm{pF}$, $C_{on}=52.6\;\mathrm{fF}$, $C_{off}=24.2\;\mathrm{pF}$ and $r_{c}=460.5$, respectively. As with the above analysis, for the constant capacitance $C_{0}$ and the difference between $A_{up}$ and $A_{dn}$, the high capacitance ratio $r_{c}$ which is greater than the conventional MEMS switch has been obtained.

## 4. Fabrication, Measurements and Discussions

### 4.1. Fabrication

**Figure 6:**
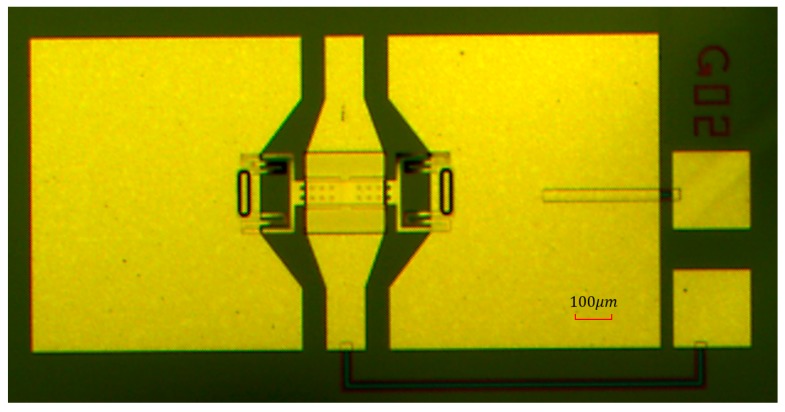
The top view photograph of the proposed high OFF/ON capacitance ratio RF MEMS switch.

The overall structure of the proposed high-capacitance-ratio RF MEMS switch was fabricated on a high resistivity silicon substrate with the thickness of 400 μm and the dielectric constant of 11.9. The SiO_2_ layer, which acts as an insulating layer, with a thickness of 0.3 μm, was formed by thermal oxidation. Then, 0.2 μm thickness of Au was deposited and patterned to define DC bias pads afterwards and to form the CPW transmission lines. Next, thin CrSi (approximately 0.05 μm) was patterned by lifting off to form the bias lines after deposition. A Si_3_N_4_ layer with thickness of 1000 Å was patterned on the top of the electrode and bias lines by plasma enhanced chemical vapor deposition (PECVD) process. 1μm Au was evaporated as the MIM floating metallic membrane. 3 μm thickness of Au, which acts as the anchors, was evaporated. Polyimide as the sacrificial layer was cut down by chemical mechanical polishing (CMP) process. The beam used 1 μm of Au. Finally, the wafer was released in a plasma dryer to avoid collapsing the membrane. The photograph of the proposed RF MEMS switch is shown in Figure 6.

### 4.2. Measurement and Results

#### 4.2.1. Insertion Loss and Isolation

The insertion loss shows the signal loss when the RF MEMS switch is in the up state (namely ON state), and the isolation indicates the signal isolation level when the RF MEMS switch is in the down state (namely OFF state). The isolation and insertion loss can be obtained by measuring the $S_{21}$ value between the input and output. With a higher insertion loss and isolation, the signal loss is less and the signal isolation level is higher.

#### 4.2.2. Capacitance Ratio

The capacitance ratio $r_{c}$ is a key parameter of the proposed RF MEMS switch. However, the OFF and ON state capacitance value of $C_{on}$ and $C_{off}$ are hard to measure. Thus, in this paper the capacitance ratio $r_{c}$ was got by S parameter extraction.

The RF MEMS switch and CPW transmission line consist of three parts and can be expressed by ABCD matrix:

(6) $$\left(\begin{array}{cc} A & B\\ C & D \end{array}\right)=M_{1}M_{2}M_{1}$$

where $M_{1}$ represents the CPW transmission line part (the ABCD matrix consists of two $M_{1}$ for the symmetry). $M_{2}$ represents the lumped parameter model of the RF MEMS. They are:

(7) $$M_{1}=\left(\begin{array}{cc} \cos\theta & jZ_{0}\sin\theta \\ j\frac{1}{Z_{0}}\sin\theta & \cos\theta \end{array}\right)$$

(8) $$M_{2}=\left(\begin{array}{cc} 1 & 0\\ Y_{2} & 1 \end{array}\right)$$

where θ is CPW transmission line electric length, $Z_{0}$ is the characteristic impedance of transmission line, $Y_{2}$ is:

(9) $$Y_{2}=\frac{1}{{\left(jwC_{s}\right)}^{-1}+jwL+R_{s}}$$

where $C_{s}$ is $C_{\mathrm{on}}$ or $C_{off}$ when the RF MEMS stays the corresponding state. The expression $L={\left(\omega C_{s}\right)}^{-1}$ is satisfied when the RF MEMS operates at the resonant frequency. $R_{s}$ is the loss resistance. $S_{21}$ parameter is:

(10) $$S_{21}=\frac{2}{A+B/Z_{0}+CZ_{0}+D}$$

**Figure 7:**
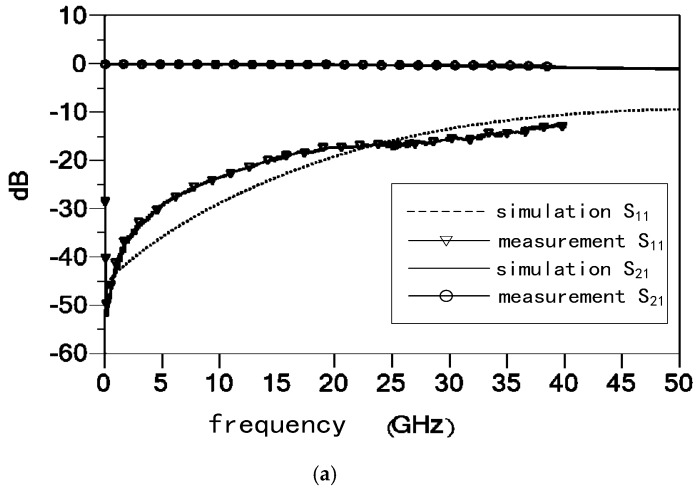

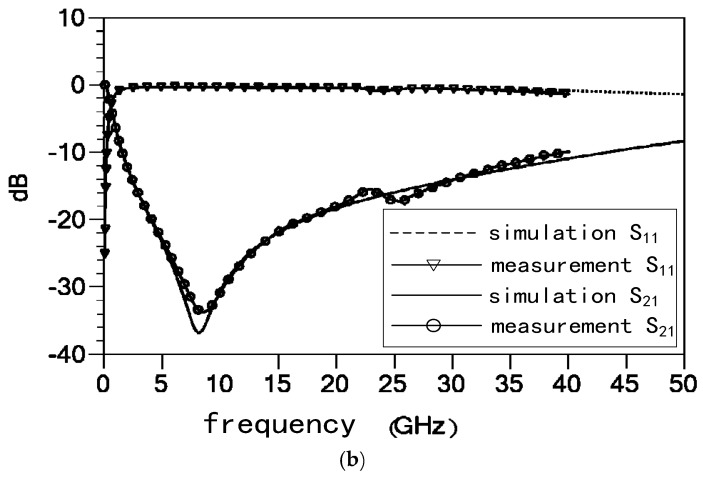
Measurement and simulation S parameters results of the proposed high-capacitance-ratio RF MEMS switch. (**a**) Up state; (**b**) Down state.

The S parameters of the proposed RF MEMS switch were measured by the network analyzer. The RF MEMS switch was fed by ground-signal-ground (GSG) probe. The measured and simulated S parameters are shown in Figure 7. Figure 7 shows the $S_{21}$ and $S_{11}$ of measured results and circuit models of MEMS switches, and the $S_{21}$ of the circuit models matches the measured results well. The measured results show that the insertion loss is better than 0.5 dB up to 40 GHz, and the isolation is more than 34 dB at the resonant frequency.

The capacitance $C_{on}$ and $C_{off}$ can be solved by using the above equations. $C_{on}=54.2\;\mathrm{fF}$, $C_{off}=20.8\;\mathrm{pF}$, L=23.6 pH, $R_{s}=0.5\;\Omega$ and $r_{c}=383.8$. The capacitance ratio $r_{c}$ is less than the calculated value; this is because the MEMS beam does not completely make contact with the CPW transmission line in the down state. The capacitance ratio of conventional MEMS switch is about 100. Hence, the capacitance ratio in this design is about three times that of the conventional design.

#### 4.2.3. Actuation Voltage

**Figure 8:**
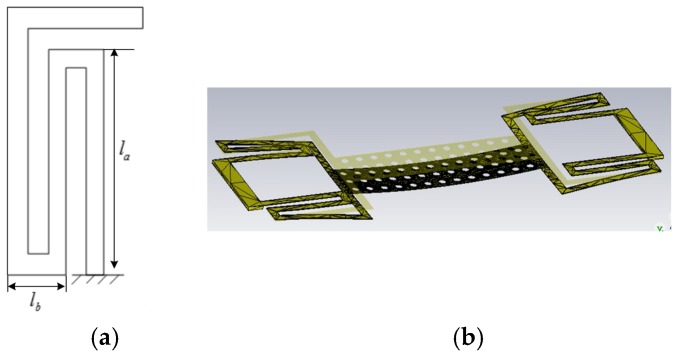
The structure of the MEMS beam. (**a**) A quarter of the beam; (**b**) Computer simulation technology (CST) simulation of the force of the switch beam.

The elastic coefficient of the proposed RF MEMS beam determined the actuation voltage. In this paper, for the symmetry of the RF MEMS beam, the elastic coefficient can be calculated by analyzing a quarter of the beam. The elastic coefficient of serpentine flexure MEMS beam can be achieved using the method described in Reference [2]. The structure of MEMS beam is shown in Figure 8. Each meander section is made of six beam segments. The actuating voltage of the can be evaluated by formula:

(11) $$V_{p}=\sqrt{\frac{2k_{e}}{\varepsilon_{0}WL_{d}}\frac{g_{0}}{3}{\left(\frac{2g_{0}}{3}+\frac{t_{e}}{\varepsilon_{r}}\right)}^{2}}$$

where *k_e_* is effective elastic coefficient, $\varepsilon_{0}$ is dielectric constant in the free space, *g*_0_ is air gap between RF MEMS switch beam and the Si_3_N_4_ dielectric when no actuating voltage is applied to the beam, $\varepsilon_{r}$ is relative dielectric constant of Si_3_N_4_ dielectric, *t_e_* is the thickness of Si_3_N_4_ dielectric layer, *W* is the width of MEMS switch beam, and *L_d_* is the length of Si_3_N_4_ dielectric, respectively. The calculated value of effective elastic coefficient *k_e_* is 19.5 N/m, and the actuating voltage $V_{p}$ is 12.6 V approximately.

However, the measurement of actuating voltage is 21.0 V, it is different from the calculation value using Equation (11). This is mainly caused by the incomplete release of polyimide and the inhomogeneity of thickness. When the manufacture process has a good release and flatness, the actuating voltage will decrease to evaluating value.

#### 4.2.4. Actuation and Releasing Time

**Figure 9:**
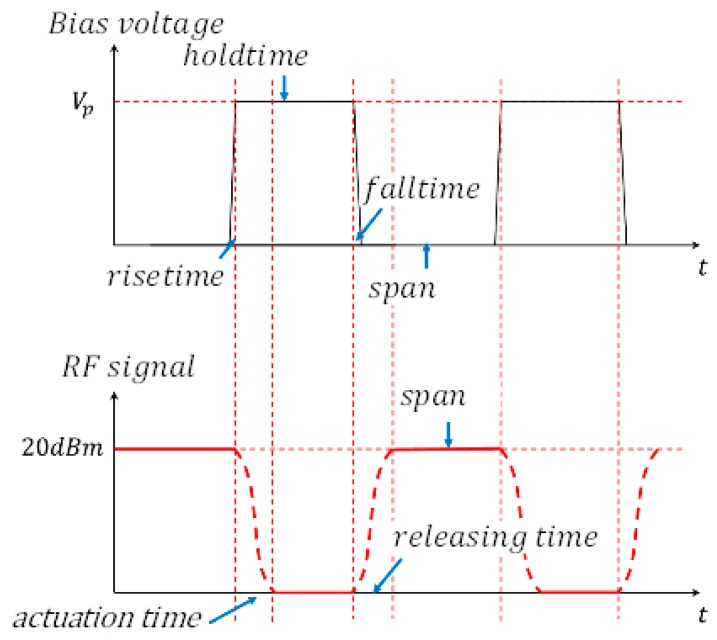
The principle of actuation time and releasing time test.

**Figure 10:**
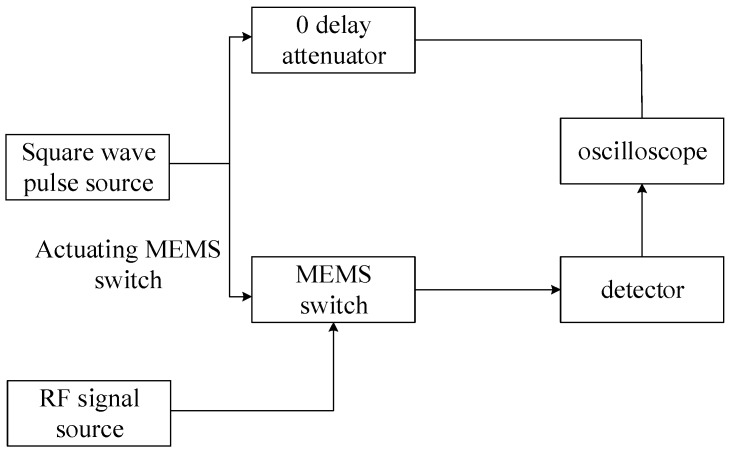
The equipment and connection.

**Figure 11:**
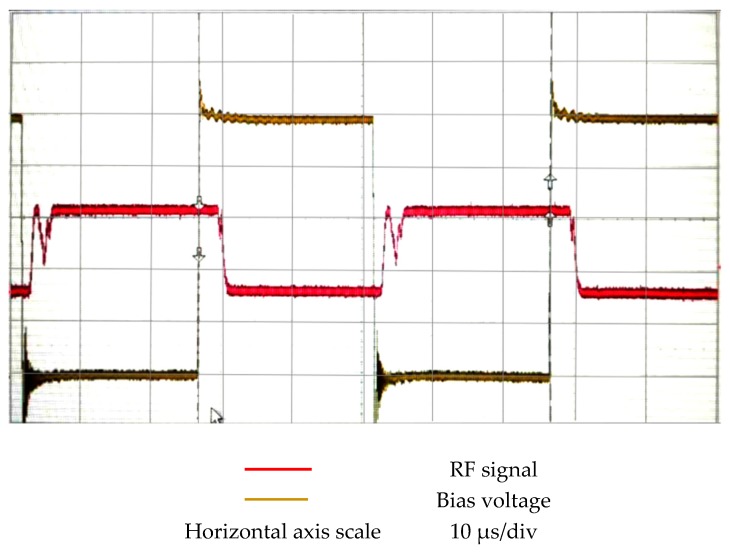
The measurement results of the actuation time and releasing time.

The actuating time of the RF MEMS switch is the time that the gap becomes zero by the actuating voltage, and the releasing time represents the time consumed on the gap release to $g_{0}$. This paper employed a method which can measure the actuation time and releasing time. The RF signal with a constant power, 20 dB·m, was applied to the RF MEMS switch; meanwhile, the RF MEMS was actuated by periodical bias voltage. The definitions of actuation time and releasing time are shown in Figure 9. The equipment and connection employed to test actuation time and releasing time are displayed in Figure 10. The periodic bias voltage has a steep rising edge and falling edge; this can guarantee the high resolution of actuation time and releasing time. The response time of the switch is shown in Figure 11. The measurement results were actuation time of 5μs, and releasing time of 6μs, respectively. The response time was less than 10 μs; this result indicates that the designed high-capacitance-ratio MEMS have rapid response speed.

During the measurement of reliability the switch was actuated with a square pulse. The switch was functional even after 10^5^ cycles (when the test was terminated for convenience).

### 4.3. Advancements

The performance comparisons of the proposed high-capacitance-ratio MEMS with generic designs are shown in Table 1. The high capacitance ratio was achieved in Reference [4] by employing dielectric materials SrTiO ($\varepsilon_{r}$ = 30–120). This paper proposes the design of MEMS switches with dielectric material of Si3N4 ($\varepsilon_{r}$ = 7.6). The compared results show that the proposed high capacitance ratio has advantages of down capacitance and capacitance ratio over those in the available literature. In addition, the proposed high-capacitance-ratio MEMS switch also has an acceptable insertion loss insulation and response time.

## 5. Conclusions

The switches presented in this work show a significant increase of capacitance ratio without taking advantage of high dielectric constant material. A high-capacitance-ratio MEMS switch with capacitance ratio 383.8 is presented for the sake of verification of the proposed method. Achieved lowest actuation voltage of the fabricated switches was 21 V. The insertion loss was better than 0.5 dB up to 40 GHz, and the isolation was more than 34 dB at the resonant frequency. Due to the excellent performances, the proposed pattern reconfigurable antenna is an excellent candidate for satellite searching, tracing, and communication systems.

## Figures and Tables

**Table 1:** Performance comparisons of the proposed high-capacitance-ratio MEMS with generic designs.

Reference	Insertion Loss (dB)	Insulation (dB)	Up Capacitance (fF)	Down Capacitance (pF)	Actuating Voltage (V)	Air Gap (μm)	Response Time (μs)	Capacitance Ratio
[4]	0.08	42	83	50	8	2.5–3.5	-	600
[7]	0.15	40	4–6	1–1.5	80	~15	1–20	250
[10]	0.35	37	51	6	117.6	2	-	22
[11]	0.2	38.5	24	1.55	12	~2	10–15	64.6
[12]	0.7	35	35	3	30	3~5	7	85.7
[13]	1.5	20	22	2.2	30	3.2	-	100
This paper	0.5	34	54.2	20.8	21	2	<10	383.8
